# Supplementary material for: Photonic neural networks at the edge of spatiotemporal chaos in multimode fibers
Source: Nanophotonics. 2025 Feb 10;14(16):2723–32. doi: 10.1515/nanoph-2024-0593 (PMC12338870; doi:10.1515/nanoph-2024-0593)
Supplement: Supplementary file 2 — Supplementary Material Details [file j_nanoph-2024-0593_suppl_002.pdf]

Supplementary Material

Bahadır Utku Kesgin, and Uğur Teğin*

Photonic neural networks at the edge of spatiotemporal chaos in multimode fibers

Supplementary Notes 1: Stability of experiment and coupled power with vortex beam

In nonlinear optical computing, the most important variable is the optical power within the system as nonlinear interactions depend on intensity. Fluctuations in the output power of the laser are expected due to non-ideal environmental factors and inevitable noise. To test the overall power fluctuations, we measured the power coupled to the fiber with a power meter before the experiments. Although the duration of the longest experiment was 6.4 hours (FashionMNIST [1]) we measured the fiber-coupled output power for 12 hours. Normalizing the output power by the maximum value of the measurements, the minimum coupled power was 0.9817. The duration of other experiments is 2.5 hours for EuroSAT [2] and 5 minutes for BreastMNIST [3].

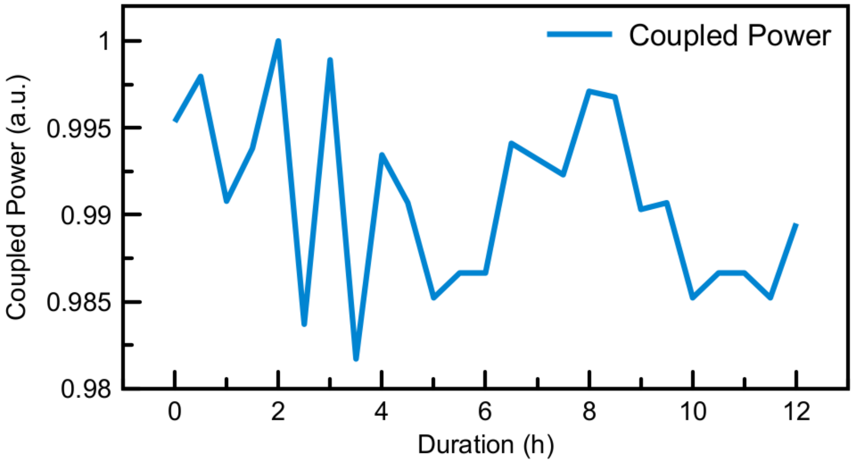

Supplementary Figure 1: Fiber coupled laser power.

Supplementary Notes 2: Preliminary tests on spatiotemporal chaotic state with vortex beam

In this study, we demonstrate an optical computing system based on the excitation of high order modes. When the initial energy of high order modes compared to fundamental modes is higher than 1/50, spatiotemporal chaos occurs, and fiber cannot converge into a stable state[4]. In high powers, the stable state refers to beam cleaning. We tested the impact of the vortex beam compared to the Gaussian beam with and without information encoding in 2kW peak power. As described in the results section, we added a blazed grating phase to prevent unmodulated zero order from entering the fiber. Due to the orbital angular momentum phase spot size of the bottle beam will be slightly larger than the regular Gaussian beam. We measured the power of fiber-coupled light with and without a vortex beam to test full coupling at 3 kW peak power. Without the vortex beam, we measured an average power of 18.4mW and with the vortex beam, we measured an average power of 18.1mW. Since we encode information in an inner square and operate at 2 kW peak power, this

***Corresponding author: Uğur Teğin**, Koç University, İstanbul, Türkiye; utegin@ku.edu.tr; https://orcid.org/0000-0002-4690-588X
Bahadır Utku Kesgin: Koç University, İstanbul, Türkiye; bkesgin22@ku.edu.tr; https://orcid.org/0009-0002-7262-3656

coupling difference is negligible. If we increase the charge of orbital angular momentum phase, the spot size will also increase. Due to this we did not increase the charge further since it will lead to loss of coupled power.

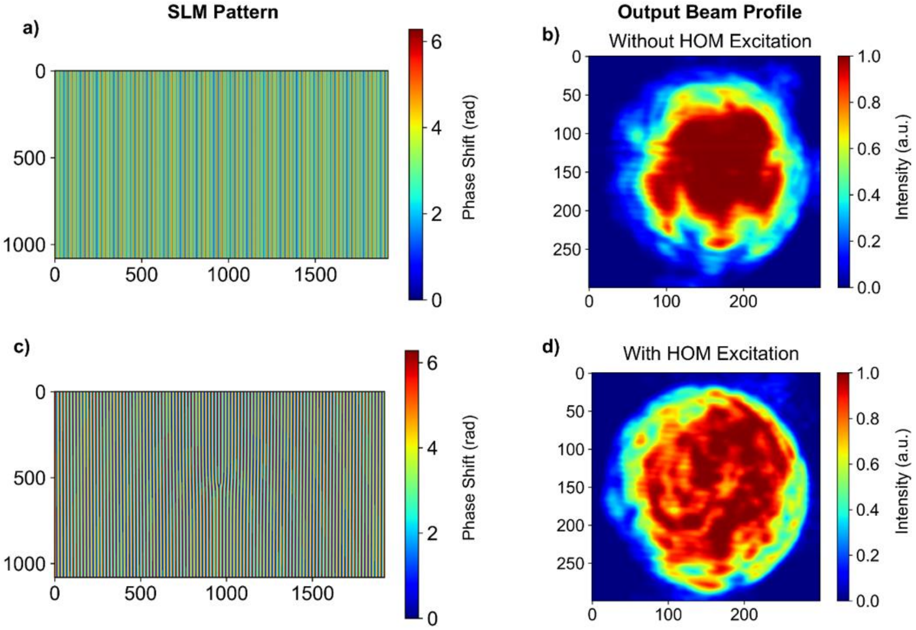

Supplementary Figure 2: Impact of vortex beams on the stability of the propagation. **a.** Regular modulation phase is applied to the input Gaussian beam. **b.** Output beam profile without high order mode excitation. **c.** The orbital angular momentum modulation phase was applied to the input Gaussian beam. **d.** Output beam profile with high order mode excitation.

Supplementary figure 2 demonstrates that without the excitation of high order modes system starts to converge to its stable state. By increasing the initial energy coupled to high order modes system cannot converge to its stable state verifying the numerical results and the presence of spatiotemporal chaos in multimodal propagation. As a final preliminary test, we sent different information patterns with vortex beams and verified our observations.

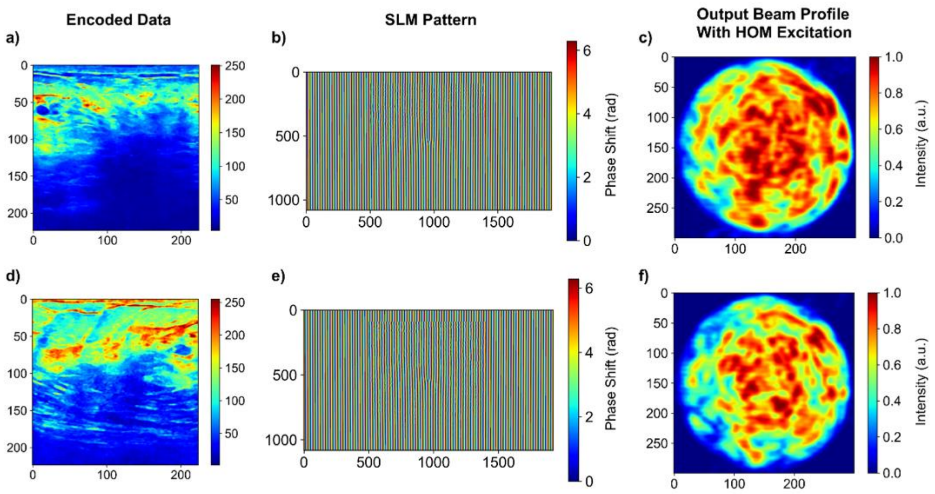

Supplementary Figure 3: Impact of vortex beams on the stability of the propagation with data. **a,d.** Encoded data in phase patterns. **b, e.** The orbital angular momentum modulation phase that was applied to the input Gaussian beam. **c,e.** Output beam profile with high-order mode excitation.

Supplementary Notes 3: Training of comparison neural networks

We train LeNet-1 and LeNet-5[5] to compare our model with convolutional neural networks that are composed of similar number of trainable parameters. We train aforementioned models by implementing their proposed architectures utilizing a computer with an RTX 4070 graphic processing unit. We train every model 300 epochs with a learning rate of 10^{-4} using ADAM optimization and Cross-entropy loss. In test set accuracy we have used the same test set as our reservoir computer.

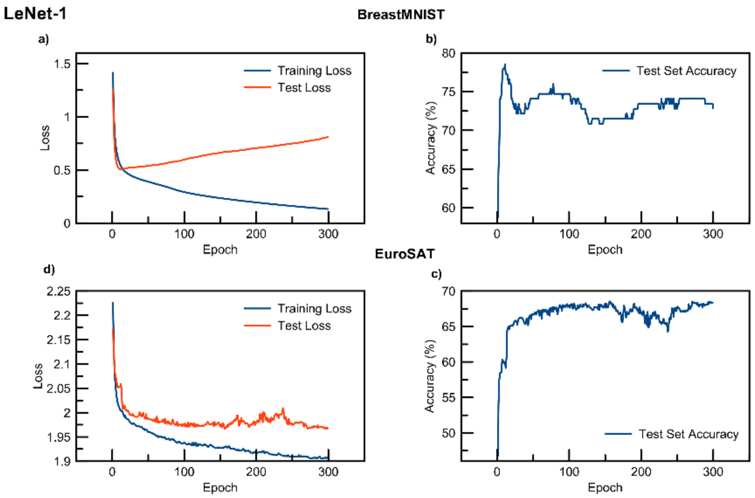

Supplementary Figure 4: Training and test loss curves and test set accuracy of LeNet-1. **a.** Training and test losses by number of epochs for BreastMNIST dataset. **b.** Test set loss by number of epochs for BreastMNIST dataset. **c.** Training and test losses by number of epochs for EuroSAT dataset. **d.** Test set loss by number of epochs for EuroSAT dataset.

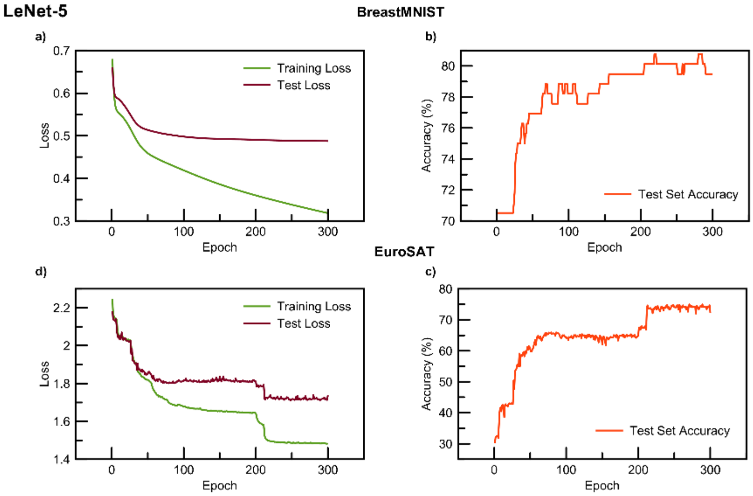

Supplementary Figure 5: Training and test loss curves and test set accuracy of LeNet-5. **a.** Training and test losses by number of epochs for BreastMNIST dataset. **b.** Test set loss by number of epochs for BreastMNIST dataset. **c.** Training and test losses by number of epochs for EuroSAT dataset. **d.** Test set loss by number of epochs for EuroSAT dataset.

Supplementary Material References

[1] H. Xiao, K. Rasul, and R. Vollgraf, “Fashion-MNIST: a Novel Image Dataset for Benchmarking Machine Learning Algorithms,” Sep. 15, 2017, *arXiv*: arXiv:1708.07747. doi: 10.48550/arXiv.1708.07747.

[2] P. Helber, B. Bischke, A. Dengel, and D. Borth, “EuroSAT: A Novel Dataset and Deep Learning Benchmark for Land Use and Land Cover Classification,” *IEEE J. Sel. Top. Appl. Earth Obs. Remote Sens.*, vol. 12, no. 7, pp. 2217–2226, Jul. 2019, doi: 10.1109/JSTARS.2019.2918242.

[3] J. Yang *et al.*, “MedMNIST v2 - A large-scale lightweight benchmark for 2D and 3D biomedical image classification,” *Sci. Data*, vol. 10, no. 1, Art. no. 1, Jan. 2023, doi: 10.1038/s41597-022-01721-8.

[4] W. He, R. Wu, W. Hong, and A. Luo, “Modal dynamics in multimode optical fibers: an attractor of high-order modes,” *Opt. Express*, vol. 29, no. 20, pp. 32682–32690, Sep. 2021, doi: 10.1364/OE.438798.

[5] Y. LeCun *et al.*, “Backpropagation Applied to Handwritten Zip Code Recognition,” *Neural Comput.*, vol. 1, no. 4, pp. 541–551, Dec. 1989, doi: 10.1162/neco.1989.1.4.541.
